# Gemfibrozil attenuates the inflammatory response and protects rats from abdominal sepsis

**DOI:** 10.3892/etm.2015.2190

**Published:** 2015-01-19

**Authors:** CARLOS R. CÁMARA-LEMARROY, FRANCISCO J. GUZMAN-DE LA GARZA, PAULA CORDERO-PEREZ, JUAN M. IBARRA-HERNANDEZ, LINDA E. MUÑOZ-ESPINOSA, NANCY E. FERNANDEZ-GARZA

**Affiliations:** 1 Department of Internal Medicine, University Hospital ‘José Eleuterio González’, Autonomous University of Nuevo León, Monterrey, Nuevo León 64460, Mexico; 2 Department of Physiology, School of Medicine, Autonomous University of Nuevo León, Monterrey, Nuevo León 64460, Mexico; 3 Liver Unit, Department of Internal Medicine, University Hospital ‘José Eleuterio González’, Autonomous University of Nuevo León, Monterrey, Nuevo León 64460, Mexico

**Keywords:** sepsis, gemfibrozil, fibrates, inflammation, rat

## Abstract

Sepsis is a serious condition characterized by an infectious process that induces a severe systemic inflammatory response. In this study, the effects of gemfibrozil (GFZ) on the inflammatory response associated with abdominal sepsis were investigated using a rat model of cecal-ligation and puncture (CLP). Male Wistar rats were randomly divided into three groups: Sham-operated group (sham), where laparotomy was performed, the intestines were manipulated, and the cecum was ligated but not punctured; control group, subjected to CLP; and GFZ group, which received GFZ prior to undergoing CLP. The groups were then subdivided into three different time-points: 2, 4 and 24 h, indicating the time at which blood samples were obtained for analysis. Serum concentrations of tumor necrosis factor-α (TNF-α), interleukin-1 (IL-1), malondialdehyde (MDA), aspartate aminotransferase (AST), alanine aminotransferase (ALT) and lactate dehydrogenase (LDH) were determined. The LDH, AST and ALT values were significantly elevated following CLP compared with those in the sham group, and GFZ treatment was able to reduce these elevations. GFZ also reduced the sepsis-induced elevations of TNF-α and IL-1. In conclusion, GFZ treatment was able to attenuate the inflammatory response associated with CLP-induced sepsis, by diminishing the release of inflammatory cytokines, thereby reducing tissue injury and oxidative stress.

## Introduction

Sepsis is a serious condition associated with great mortality, characterized by an infectious process that induces a severe systemic inflammatory response. The physiopathology of sepsis is well understood, and mediators such as pro-inflammatory cytokines, reactive oxygen species, nitric oxide, toll-like receptors and transcription factors such as nuclear factor κB (NF-κB) play central roles in regulating the immune response responsible for tissue injury and circulatory collapse (1,2). These events lead to hypotension, increased capillary permeability, multiple organ failure and mortality.

Although various therapeutic strategies have been implemented with the aim of reducing sepsis-associated morbidity and mortality, the cornerstone of treatment remains prompt antibiotic use and hemodynamic management (3). The use of immunomodulators, such as activated protein C, has been evaluated and early results were promising (4). However, a recent trial did not find any clinical benefit with the use of this drug (5), thus questioning the use of drugs that attempt to modulate the inflammatory response. Various drugs with pleiotropic immunomodulatory properties are currently being studied, and this remains an active field of research (6).

Fibrates such as gemfibrozil (GFZ) and fenofibrate are drugs commonly used in the management of dyslipidemia. They are ligands for peroxisome proliferator-activated receptor α (PPAR-α), receptors that belong to the steroid nuclear receptor family and that participate in lipid peroxidation, the cell cycle and fatty acid synthesis. Potent immunomodulatory effects have been associated with fibrates (7). Fibrates have shown the ability to reduce the expression of inflammatory genes in endothelial cells, the release of pro-inflammatory cytokine such as interleukins (ILs) and tumor-necrosis factor-α (TNF-α), and the transcription of NF-κB in experimental studies (8,9). Fibrates have also been shown to reduce IL-1 and C-reactive protein in humans (10). Fibrates also possess antioxidant effects, and regulate the activation and function of inflammatory cells (11,12). These effects are independent of the lipid-lowering properties of fibrates.

The anti-inflammatory effects of fibrates have been tested in various experimental models of tissue and organ injury, showing beneficial results (reduced injury and inflammation) in models of autoimmune encephalomyelitis (13), ischemia-reperfusion injury (14) and alcohol-induced hepatotoxicity (15), among others. Considering the wide ranging immunomodulatory effects of fibrates, some of which overlap with the physiopathology of sepsis, the use of fibrates emerges as an interesting option.

## Materials and methods

### Animals

Animal procedures were performed in accordance with the proper use and care of laboratory animals, approved by the ethics committee of the University Hospital ‘José Eleuterio González’ (Monterrey, Mexico). Experiments were performed on 45 male Wistar rats weighing 200–250 g (Vivarium of the Department of Physiology, Autonomous University of Nuevo León, Monterrey, Mexico). Animals were maintained under standard conditions, including a stable room temperature (24±3°C), a 12 h light/12 h dark cycle, and had access to commercial rat pellets and water *ad libitum*.

### Cecal ligation and puncture model (CLP) (16)

After fasting for 12 h, the rats underwent ketamine/xylazine anesthesia (Anesket; Pfizer Inc., Mexico City, Mexico) at a dose of 50/10 mg/kg, intraperitoneally (i.p). Animals were placed under a heating lamp in order to preserve a core body temperature of 37°C. A midline incision exposed the intestines and the cecum was ligated (using 2-0 silk sutures) immediately proximal to the ileocecal valve causing a 50% obstruction and allowing for permeability. Two through-and-through punctures using a 18-gauge needle were performed at the anti-mesenteric side of the cecum. Light pressure was applied to confirm that fecal matter could emerge into the peritoneal cavity. The cecum was returned to its site, the wound was closed and 3 ml/100 g saline was administered subcutaneously.

The rats were randomly divided into three groups (n=15 per group): i) Sham-operated group (sham), where laparotomy was performed, the intestines were only manipulated and the cecum was ligated but not punctured. ii) Control group, subjected to CLP as described above. iii) GFZ group that received GFZ (Pfizer Inc.) prior to undergoing CLP. A dose of 100 mg/kg GFZ was chosen based on dose-response experiments and was administered 24 h and immediately prior to surgery. This dose has been shown to decrease oxidative stress in rodent models (11). The groups were then subdivided into three different time-points: 2, 4 and 24 h. These time-points indicated the time after CLP when samples were obtained for analysis.

### Serum analysis

Blood samples obtained following CLP were used to determine the serum levels of aspartate aminotransferase (AST), alanine aminotransferase (ALT) and lactate dehydrogenase (LDH) by standard biochemical automated methods (Vitros Chemical Products; Johnson & Johnson, New Brunswick, NJ, USA), using commercially available kits and DT6011 and DTSC11 analyzers (Vitros Chemical System; Johnson & Johnson). The serum concentrations of TNF-α and IL-1 were determined using a rat ELISA kit (PeproTech, Mexico City, Mexico). Lipid peroxidation, expressed as the malondialdehyde (MDA) level, was assessed by the thiobarbituric acid reactive substances (TBARS) method using a TBARS colorimetric assay kit (Cayman Chemical Company, Ann Arbor, MI, USA). The level of LDH was only evaluated at the 24-h time-point after CLP.

### Survival

To assess survival, additional groups of rats (n=10 per group) underwent the same study protocol as those in the control and GFZ groups, and the rats were left to recover following the surgery. The animals were observed for a period of five days and the mortality rate was recorded.

### Statistical analysis

SPSS statistical software, version 11.0 (SPSS Inc., Chicago, IL, USA) was used to analyze data using one-way analysis of variance (ANOVA) and with LSD post-hoc test (when data were found to be normally distributed) and Kruskal-Wallis test (when data were not normally distributed) so as to evaluate comparisons between groups, and differences between groups, respectively. The survival curves were determined using the Kaplan-Meier method and the log-rank test was used to compare the curves. All values are expressed as mean ± standard deviation (SD) and P<0.05 was considered to indicate a statistically significant result.

## Results

### ALT and AST

AST and ALT values were significantly elevated following CLP compared with those in the sham group, peaking at 4 h for AST and 24 h for ALT (Table I). GFZ treatment was able to reduce the elevations in AST and ALT, with its effects reaching statistical significance at 2 and 4 h in the case of AST and at 4 h in the case of ALT. At 24 h after CLP, LDH levels were significantly elevated, and GFZ treatment was also able to attenuate this increase.

### Serum cytokine levels

**Figure 1:**
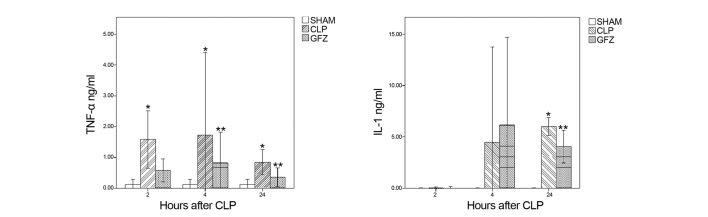
Levels of inflammatory cytokines. ^*^P<0.05 vs. SHAM; ^**^P<0.05 vs. CLP. TNF, tumor necrosis factor; IL-1, interleukin-1; GFZ, gemfibrozil; CLP, cecal ligation and puncture.

Levels of TNF-α were significantly elevated in all time-points in the CLP group as compared with those in the sham controls (Fig. 1). TNF-α levels peaked at 4 h and remained elevated even at 24 h. GFZ treatment resulted in significantly lower levels of TNF-α at 4 and 24 h after CLP. In the case of IL-1, there was no difference between the groups at 2 h, where levels were undetectable by the assay. At 4 and 24 h, the IL-1 levels were elevated; however, statistical significance was reached only at 24 h. At this time-point, GFZ reduced IL-1 levels significantly (Fig. 1).

### MDA

There were two identifiable time-points of increased MDA levels in the CLP group, at 2 and 24 h. However, no difference was found between the CLP and GFZ groups (data not shown).

### Survival

By 48 h, five rats in the CLP group and three in the GFZ group had died. At 5 days, seven rats in the CLP group and five rats in the GFZ group had died. The difference in survival between groups did not reach statistical significance (P=0.1).

## Discussion

PPARs have been studied in models of sepsis, and PPAR-γ agonists have been found to be effective in reducing the severity of endotoxic shock (17). A recent study showed that PPAR-α expression was decreased in patients with septic shock and that the values correlated with the severity of disease; in addition, in the CLP model, knockout mice lacking PPAR-α had decreased survival rates compared with wild-type animals (18). Fenofibrate treatment has been shown to reduce endothelial dysfunction and endothelial cell injury in a rabbit model of endotoxemia by lipopolysaccharide (LPS) injection (19). Improved endothelial-dependent relaxation and decreased monocyte tissue factor expression were observed. Another model of LPS-induced endotoxemia in rats also demonstrated reductions in myocardial contractility depression and in TNF-α levels after fenofibrate treatment (20). These results are in contrast to those in another study where dietary fenofibrate caused increased levels of TNF-α and mortality in endotoxemic wild-type mice, while showing lower TNF-α levels in PPAR-α knockout mice (21). Using a physiologically relevant model of abdominal sepsis, in the present study it was demonstrated that GFZ pretreatment reduced the inflammatory response associated with abdominal sepsis. GFZ had immunomodulatory effects, reducing the circulating levels of TNF-α, IL-1 and MDA, as well as markers of tissue injury such as AST and ALT. Differences between the LPS injection and CLP models could be relevant in explaining these inconsistent results, although the present study would support those showing a beneficial effect of PPAR-α ligands such as fibrates, despite only a trend towards a survival benefit being observed.

Inflammatory cytokines play an important but complex role in sepsis (1,2,22). Some of the mechanisms by which fibrates might suppress inflammatory cytokines are beginning to be uncovered. In a study with LPS-activated cells, PPAR-α ligands, including fibrates, were able to inhibit NF-κB DNA binding activity in astrocytes, leading to a modulation of the expression of inflammatory genes responsible for the production of cytokines such as IL-1 and TNF-α (23). Similar results were obtained in LPS-stimulated cardiac myocytes (24), and in injured endothelial cells (9). Clinical studies have shown that GFZ reduces TNF-α production in human peripheral blood mononuclear cells (25), and fibrates reduce IL-1 in human whole blood stimulated by endotoxin (26). Fibrates dose-dependently inhibit cytokine production (ILs, TNF-α and interferons) in activated T cells, probably via the inhibition of transcription factors associated with the inflammatory response, such as activator protein-1, c-Jun NH2-terminal protein kinase and P38 mitogen-activated protein kinase (27). The expression of IL-1 receptor antagonist, which is acutely stimulated by LPS treatment in the liver, can also be induced by PPARα (28). The switching of T cells from Th1 to Th2 profiles by GFZ is responsible for the tissue protection afforded against experimental encephalomyelitis in rats (12). Interestingly, the majority of studies use LPS, a key mediator of the sepsis inflammatory cascade, as a stimulant for cytokine production. In one study, using LPS-stimulated airway inflammation, PPAR-α^−/−^ mice exhibited increased neutrophil infiltration and TNF-α production compared with that in PPAR-α^+/+^ mice, and fenofibrate was able to reduce TNF-α production in wild-type mice (8).

NF-κB is a key mediator of the sepsis inflammatory cascade. NF-κB activity is markedly increased in every organ studied, both in human and experimental models of septic shock, and greater levels of NF-κB activity are associated with a higher rate of mortality and worse clinical outcome in septic patients (29). The inhibition of NF-κB, and of the subsequent overproduction of inflammatory cytokines such as TNF-α, appears to be a crucial step in the immunomodulatory effects of PPAR-α activators. Cultured injured endothelial cells show activation of NF-κB that can be inhibited by incubation with fenofibrate (9). Fibrates were able to induce the expression of the inhibitory protein IκBα in human aortic smooth muscle cells as well as in primary human hepatocytes, providing a possible mechanism for NF-κB inhibition (30). Another study has also linked TLR to PPAR-α signaling. The PPARα agonist fenofibrate was found to reduce inflammatory cytokines and inflammation by antagonizing LPS-mediated inflammatory responses in vascular smooth muscle cells through a mechanism involving TLR-4 (31).

Marked oxidative stress results from the initiation of the inflammatory response in sepsis, and it initiates changes in mitochondrial function that may result in organ damage (32). Sepsis suppresses free fatty acid oxidation with the result of increased circulating fatty acids, through LPS-induced suppression of PPAR-α (33). Additionally, LPS induces the production of MDA and depletes catalase and superoxide dismutase in inflammatory cells (34). A previous study found elevations in MDA and glutathione depletion following CLP in rats (35). Fibrates are known to diminish MDA production in diabetic rats (36), and following severe liver ischemia-reperfusion injury in rats, fibrate pretreatment is able to reduce elevations in MDA and depletion of endogenous antioxidants (37). Elevations in MDA after CLP were observed in the present study, but only in untreated animals. No difference in MDA levels was identified between the CLP and GFZ group.

Although the inhibition of single cytokines or the addition of antioxidants has not shown value in the treatment of sepsis (38,39), the use of immunomodulators with pleiotropic effects has been continuously studied (6). Lipid-lowering drugs such as statins are currently thought to be prime candidates as adjunct treatments in sepsis (40), and clinical trials are underway. In this study it was found that another class of lipid-lowering drugs, fibrates, also have immunomodulating properties and could also be of value. The use of these drugs, alone or in combination, warrants further study.

## Figures and Tables

**Table I:** Serum levels of ALT, AST and LDH.

Group	AST (UI)	ALT (UI)	LDH (UI)
Sham	109.0±10.6	15.8±5.2	537.0±141.4
CLP 2 h	271.4±58.7^a^	27.4±10.2	
CLP 4 h	293.8±113.9^a^	39.8±16.3	
CLP 24 h	200.8±16.6^a^	44.0±2.7^a^	1983.4±530.7^a^
GFZ 2 h	175.8±69.9^b^	19.0±11.6	
GFZ 4 h	204.6±28.8^b^	19.4±5.1^b^	
GFZ 24 h	172.2±33.9	37.4±8.3	1057.4±403.2^b^

Values are expressed as mean ± standard deviation. ALT, alanine aminotransferase; AST, aspartate aminotransferase; LDH, lactate dehydrogenase;

a P<0.05 vs. Sham;

b P<0.05 vs. CLP.

GFZ, gemfibrozil; CLP, cecal ligation and puncture.
